# Calculating Polygenic Risk Scores (PRS) in UK Biobank: A Practical Guide for Epidemiologists

**DOI:** 10.3389/fgene.2022.818574

**Published:** 2022-02-18

**Authors:** Jennifer A. Collister, Xiaonan Liu, Lei Clifton

**Affiliations:** Nuffield Department of Population Health, University of Oxford, Oxford, United Kingdom

**Keywords:** polygenic risk score, UK biobank, genetic risk score, worked example, polygenic score

## Abstract

A polygenic risk score estimates the genetic risk of an individual for some disease or trait, calculated by aggregating the effect of many common variants associated with the condition. With the increasing availability of genetic data in large cohort studies such as the UK Biobank, inclusion of this genetic risk as a covariate in statistical analyses is becoming more widespread. Previously this required specialist knowledge, but as tooling and data availability have improved it has become more feasible for statisticians and epidemiologists to calculate existing scores themselves for use in analyses. While tutorial resources exist for conducting genome-wide association studies and generating of new polygenic risk scores, fewer guides exist for the simple calculation and application of existing genetic scores. This guide outlines the key steps of this process: selection of suitable polygenic risk scores from the literature, extraction of relevant genetic variants and verification of their quality, calculation of the risk score and key considerations of its inclusion in statistical models, using the UK Biobank imputed data as a model data set. Many of the techniques in this guide will generalize to other datasets, however we also focus on some of the specific techniques required for using data in the formats UK Biobank have selected. This includes some of the challenges faced when working with large numbers of variants, where the computation time required by some tools is impractical. While we have focused on only a couple of tools, which may not be the best ones for every given aspect of the process, one barrier to working with genetic data is the sheer volume of tools available, and the difficulty for a novice to assess their viability. By discussing in depth a couple of tools that are adequate for the calculation even at large scale, we hope to make polygenic risk scores more accessible to a wider range of researchers.

## 1 Introduction

**FIGURE 1:**
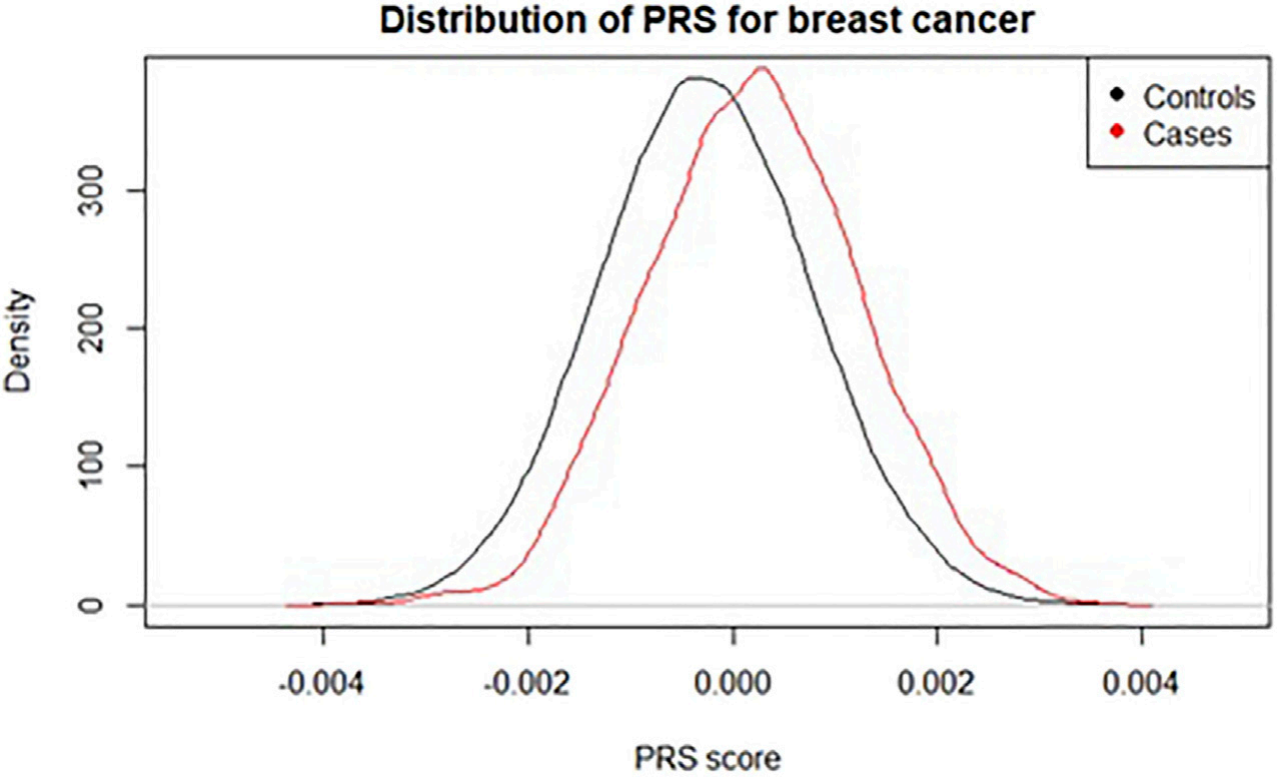
Distribution of a polygenic risk score for breast cancer among individuals with and without registry-verified breast cancer events in UK Biobank (4,789 cases). Score used was 313-SNP PRS from (Mavaddat et al., 2019).

A polygenic risk score (PRS), sometimes called polygenic score (PGS) or genetic risk score (GRS), is an estimate of an individual’s genetic risk for some trait, obtained by aggregating and quantifying the effect of many common variants (usually defined as minor allele frequency ≥1%) in the genome, each of which can have a small effect on a person’s genetic risk for a given disease or condition. A PRS is typically constructed as the weighted sum of a collection of genetic variants, usually single nucleotide polymorphisms (SNPs) defined as single base-pair variations from the reference genome. The resulting score is approximately normally distributed in the general population, with higher scores indicating higher risk (Figure 1).

The basic equation for the PRS of an individual j is:

Eq. (1): Standard equation to calculate a weighted polygenic risk score

$$PRS_{j}=\overset{N}{\underset{i}{\sum }}\beta_{i}*dosage_{ij}$$

where N is the number of SNPs in the score, $\beta_{i}$ is the effect size (or beta) of variant i and $dosage_{ij}$ is the number of copies of SNP i in the genotype of individual j .

The effect sizes, or betas, are often obtained from a genome-wide association study (GWAS) known as the “base” data (see Table 1: Glossary), wherein each genetic marker in turn is tested for association with the trait/disease of interest, and effect sizes are estimated.

**TABLE 1:** Glossary.

Term	Meaning
Allele	An alternative form of a genetic variant
Alternate id	In the UK Biobank multi-allelic SNPs are represented as multiple SNPs with different alleles but the same rsID and same position on the chromosome. In order to have a unique identifier for each SNP, an “alternate_id” was created that is typically the rsID, chr:pos or Affymetrix identifier followed by the reference and alternate alleles
Base data	Typically GWAS summary statistics containing SNP identifiers, risk alleles and effect sizes
Genome Build	The genome build is a common “reference genome” developed by combining the sequences most commonly observed across available individual genomes to create a representative genome against which individual genomes can be compared
Genotype data	Genotyping is the identification of the genetic variants in the DNA of an individual. This is typically done using arrays or chips, which contain probes that target specific locations in the DNA. These locations contain known variants of interest—so genotyping is good at identifying which known variants a person has, but not at finding new variants
Genotype Imputation	Genotype imputation uses a reference panel to estimate genotypes at locations that were not directly called by statistical inference
Heritability	Heritability is the amount of observable (phenotypic) variation among individuals of a population that is due to genetic variation between the individuals
Linkage Disequilibrium (LD)	Linkage disequilibrium (LD) is a measure of the correlation between neighbouring genetic variants that are more likely to be inherited together because of their physical proximity, leading to association within a population
Locus	Physical location of a gene or DNA polymorphism on a chromosome (plural “loci”)
Multi-allelic SNPs	When there is more than one possible variant nucleotide (in addition to the reference) at a location, then we say this location is “multi-allelic”
Next generation sequencing	Sequencing enables the exact sequence of bases in a length of DNA to be determined. This technique can be used on targeted areas such as the exome, although it is becoming increasingly cost effective to do whole genome sequencing
Phenotype	The phenotype of an organism is its observable characteristics, for example its physical appearance
rsID	The rsID for a SNP is the unique RefSNP ID number identifying the “reference SNP cluster” containing this SNP in dbSNP. This cluster contains all SNPs that map to the same location on the genome
	Since genome assemblies are still a work in progress, occasionally there will be changes that alter our understanding of where a refSNP is located, so that it may co-locate with another existing refSNP. In these cases, the higher refSNP number is retired and all SNPs are reassigned to the refSNP with the lower number
Single Nucleotide Polymorphism (SNP)	A single nucleotide polymorphism (or single nucleotide variant) is a location on the genome where a single DNA nucleotide that differs from that in the reference genome has been identified
Target data	The data in which the PRS is developed, using effect sizes from the base data. Multiple PRS may be calculated, using different thresholds for association, and the one with best performance is selected
Validation data	The data in which the PRS is calculated and used in analyses. These analyses may validate the association between the PRS and the trait of interest

In more advanced methods of PRS development, “target” data might be used to tune parameters or perform model selection (Ma and Zhou, 2021). These approaches include the construction of multiple PRS based on different threshold values for SNP association with the trait of interest, the shrinkage of betas, and adjustment for linkage disequilibrium using techniques such as pruning and clumping (Choi et al., 2020).

Once a PRS has been developed, it is important for the association between the PRS and the trait of interest to be replicated in an independent sample, referred to as “validation” data. This is done to guard against overfitting, which can lead to inflated estimates. The PRS can then be calculated in other data-sets and used for a wide range of analyses (Lewis and Vassos, 2020; Wray et al., 2021).

There is particular interest in adding PRS to existing risk prediction models (Elliott et al., 2020; Inouye et al., 2018; Lee et al., 2019; Sun et al., 2021), which could allow them to be incorporated into clinical guidelines, enabling clinicians to identify individuals who may be at higher risk of a given condition, or who may benefit from more aggressive treatment to manage the condition.

There has also been increasing use of PRS in Mendelian Randomisation to establish the causal effect of risk factors on clinical outcomes, mainly due to simplicity of use, increased power and avoidance of weak instrument bias (MV et al., 2015; Gajendragadkar et al., 2021; Zekavat et al., 2021).

As increasingly many PRS are developed, initiatives such as the Polygenic Score Catalog^1^ and Cancer PRS-Web^2^ have begun to host and curate the metadata required to calculate the scores, making them more accessible for future research (Fritsche et al., 2020; Lambert et al., 2021). Despite this, it seems more common for new scores to be developed, offering only minimal improvements in population level risk prediction, than for existing scores to be used in further analyses.

In this paper, we outline the necessary considerations when selecting an existing PRS from the literature for use in new analyses, including discussion of the information required for the calculation to be reproducible. We provide a step-by-step walkthrough of how to calculate an existing PRS in an independent dataset, from extracting SNPs to the necessary quality control (QC) checks that should be performed prior to calculating the PRS. We focus in particular on imputed data, using UK Biobank v3 imputed data (March 2018) as an example, and we consider only SNPs on autosomes.

After discussing the various steps required to obtain and calculate a PRS, we present a worked example using a PRS for LDL-Cholesterol (LDL-C) and a brief discussion of the statistical considerations when including a PRS in a model. Detailed code examples are provided in the online materials^3^ on GitHub, along with notes on technical considerations.

## 2 Materials and Methods

### 2.1 Software Considerations

Genetic data can be stored in a range of different formats, and due to the large size of the data it is often compressed to save space, resulting in files that are not directly human-readable and require dedicated software tools or packages. Many such genetic software are designed to run on Linux and in this paper we will assume access to a Linux system with adequate storage space for the data.

Our example data, the UK Biobank v3 imputed data, is made available in BGEN v1.2 format (Band and Marchini, 2018) which is the format output by the IMPUTE imputation software (Marchini and Howie, 2010). There are a range of software tools that can be used to read and manipulate this data, and deciding which to use is a combination of computation time, software compatibility and personal preference. In this paper we will focus on three: bgenix,^4^ QCTOOL v2^5^ and PLINK 2^6^ (Chang et al., 2015; Band and Marchini, 2018), summarized in Table 2.

**TABLE 2:** Comparison between genetic software for various usages.

	Genetic software		
Usage	bgenix	QCTOOL	PLINK
Extract SNPs	Yes, very quickly, although can only specify up to 9,980 SNPs by chromosome and position identifier	Yes, and has useful wildcard feature to extract from all chromosome files in one step, but slow	Yes, have to extract per chromosome, slow for BGEN data as it has to auto-convert the entire file not just the required SNPs
Conduct QC	No	Yes, it computes summary statistics but filtering has to be done in a separate step, and with additional tools (such as awk or R)	Yes, fast, it can compute summary statistics and apply filtering. Not all commands are suitable for use on imputed data
Compute PRS	No	Yes but poorly documented	Yes, with many options

Bgenix is a utility that was developed alongside the BGEN file format to index and retrieve subsets from the .bgen data files. The accompanying cat-bgen utility can be used to concatenate BGEN files.

QCTOOL v2 was the tool used by UK Biobank to generate the minor allele frequency and imputation information metrics released alongside the imputed data. It can be used to produce per-SNP and per-sample summary statistics, and perform filtering of the dataset. However, it can be slow to run for larger datasets.

A more scalable alternative is PLINK 2 (Chang et al., 2015), which we recommend for the routine quality control (QC) process described in this paper. A selection of PLINK 2 commands useful for such QC are summarized in Table 3. While PLINK 1.9 has a similar feature set and could also be used, it does not directly support the BGEN v1.2 file format, and so an interim conversion step would be required.

**TABLE 3:** PLINK 2 commands for summary statistics and filtering.

Function	Summary statistics	As exclusion criteria	
		Option	Meaning
Allele frequency	--freq	--maf [threshold]	Include SNPs with MAF above [threshold] (default = 0.01)
SNP call rate	--missing	--geno [threshold]	Exclude SNPs with missing call rates exceeding the [threshold] (default = 0.1)
Filter SNPs		--exclude [file]	Exclude SNPs listed in [file]
Filter samples		--keep [file]	Retains only the samples listed in [file], all others are excluded
HWE	--hardy	--hwe [threshold]	Exclude SNPs with *p*-values below [threshold]
Linkage Disequilibrium (LD)	--r2*	--indep-pairwise [window][step][threshold]	Pruning with a [window] size, sliding across the genome with [step] size at a time and filter out any SNPs with LD r^2^ higher than [threshold]

* Command in PLINK 1.9.

In this paper we demonstrate the actual calculation of the PRS in PLINK 2, but it is numerically straightforward and can be computed in any scripting language such as R if sufficient computer memory is available. Dedicated PRS tools like PRSice-2 (Choi and O’Reilly, 2019) can also be used, but these were designed for those wishing to develop a new PRS from scratch, offering more complex functionalities and assuming a level of domain expertise that may be off-putting for a beginner/casual user.

### 2.2 Choosing a Polygenic Risk Score

In order to include a polygenic risk score in analyses, the first step is to select an existing PRS for the phenotypic trait or outcome of interest. PRS are sometimes made available in the supplementary materials of the papers where they are derived, but are increasingly being made available in online repositories such as the PGS Catalog (Lambert et al., 2021), which improve discoverability with the intention of improving the reproducibility of genetic research.

#### 2.2.1 Outcome

The research objective is the first consideration when choosing a PRS. Since any given PRS is associated with a single phenotypic trait (e.g., height, blood pressure) or medical condition/outcome (e.g., breast cancer), when choosing a PRS for use in analysis it is important to select a score that has been derived for an appropriate trait or condition.

When attempting to replicate (or validate) the association found between some given PRS and a trait/outcome then it is important to understand exactly how this trait/outcome was defined in the development of the PRS, as it will need to be defined as similarly as possible within the validation dataset. For measured traits (e.g., cholesterol), attention to units (e.g., mg/dL or mmol/L) and whether adjustments have been made for subgroups (e.g., correcting cholesterol for statin users) are typically required to produce reliable results.

An alternative objective could be to investigate whether a PRS for a trait (for example a measured biomarker such as cholesterol) is associated with an outcome linked with that trait (such as heart disease).

#### 2.2.2 Performance

When going to the trouble of including a PRS in analyses, ideally it should be one that provides as much additional information as possible. The performance of a PRS can be measured in a variety of ways - for example, one could consider the risk ratios between top and bottom percentiles of the PRS and the outcome of interest—and its stated performance should be evaluated in the context of the research goals.

Metrics commonly used to evaluate a PRS include the pseudo-R^2^, which indicates the amount of phenotypic variance explained by the PRS (Lee et al., 2012), the Brier score, and the area under the ROC curve (AUC). Some PRS repositories are starting to make this information available alongside the scores to facilitate comparison (Fritsche et al., 2020; Becker et al., 2021).

Larger base/target datasets give more power to detect association of SNPs with the trait of interest, and have been shown to yield scores with higher predictive capability (Lello et al., 2019). In addition, it has been found that aggregating SNPs that are not themselves associated with a trait at a statistically significant *p*-value threshold can still result in a significantly associated score (Agerbo et al., 2015), meaning that PRS are getting larger—some contain hundreds of thousands, or even millions of SNPs. While a large PRS including many SNPs contains more information and is likely to have better performance than a smaller PRS, there are diminishing returns here and access to computational resources may impose a practical limit on the size of PRS used.

PRS will perform best in populations of the same ancestry as those in which they were derived (Duncan et al., 2019). This is particularly important if the analysis data contains primarily non-White individuals, as although there is ongoing effort to increase the diversity of genetic data, currently most available PRSs are for individuals of White ethnicity. If the analysis population contains a mixture of ancestries we recommend a sensitivity analysis in a subpopulation with genetic ancestry as similar as possible to that in which the PRS was derived.

#### 2.2.3 Technical Considerations

It is important to avoid sample overlap between the data in which the PRS was developed (base and target), and the data in which the PRS will be used in analyses. If the same individuals are present across these datasets this can inflate the observed association between the PRS and the trait of interest—this can also occur if the datasets contain closely related individuals.

Since it may not be possible to access raw genetic data from the base/target datasets to check for duplicate or related individuals directly, we recommend that the datasets in which potential scores were developed are reviewed in order to select one where there are unlikely to be duplicated or related individuals in the intended validation data.

Finally, if the genomic positions in the GWAS where the SNPs were identified were not assigned on the same genomic build as the intended analysis data then additional software tools, such as LiftOver (Hinrichs et al., 2006), may be required to standardise this.

#### 2.2.4 Information Needed From the Original Polygenic Risk Score

At a minimum, the information needed to replicate a PRS is:

• The list of SNPs included in the score. These may be given as “dbSNP Reference SNP numbers” (refSNP or rsID), or as base-pair positions on a chromosome.
• The effect (and preferably also the non-effect) allele for each SNP.
• The effect size (weighting) for each SNP for the condition of interest.
• The genome build

These could be the raw results from a GWAS filtered to SNPs of interest, or may have had further PRS development techniques applied.

The effect size may be given as a beta (weighting) or as an Odds Ratio (OR) or Hazard Ratio (HR), depending on the original analysis and how the authors chose to present the score. It is important to understand the form the weights are provided in to know if any transformation is necessary, and how to interpret the resulting PRS—for example, OR and HR will need to be log-transformed to obtain the weights for use in the PRS calculation.

Sometimes additional information such as the effect allele frequency (EAF) is also provided. Ensuring that the allele frequencies in the validation data are consistent with those observed in the base/target data is a good check to perform when such data are available, and it can give greater confidence when dealing with ambiguous SNPs. We will discuss this further in Section 2.4.

When accessing a PRS through an online repository such as PGS Catalog then they may have a schema^7^ detailing the possible columns of information available about the score, and will have ensured uniform headings across scores.

### 2.3 Extracting SNPs

As we mentioned briefly in Section 2.1, the data we are discussing in this paper is UKB v3 imputed data, which contains ∼93M autosomal variants for ∼500,000 samples. The data is made available in BGEN v1.2 files, a binary version of the “Oxford” .gen and .sample file format, where trios of genotype probabilities for each SNP are stored in the .bgen file with a corresponding .bgen.bgi index file, and data about the individuals is stored in a .sample file providing participant IDs unique to each application. The genetic data is split by chromosome in files ranging from 40 to 200 GB.

When choosing which software tool to use to extract specific SNPs from the bulk genetic data, two main considerations are speed and compatibility with the data format. While PLINK 2 has support for BGEN v1.2 format, in order to extract a given list of SNPs, it will first auto-convert the entire data file to PLINK 2 binary format (.pgen, .pvar, .psam). This can be time-consuming considering the large size of UKB imputed data and is not lossless—PLINK 2 collapses the trios of raw genotype probabilities into single dosages according to a given threshold value (see Section 2.6.2 for more information).

For this reason we recommend bgenix, which was designed for use on BGEN format data and makes use of a SQLITE index file (.bgen.bgi) to quickly filter the required SNPs from the raw UKB imputed data files. Unfortunately one current limitation of bgenix is that while any number of SNPs can be specified by rsID, it is only possible to specify up to 9,980 distinct SNPs by chromosome and position in one command.

Due to differences in genotyping arrays, sometimes some of the SNPs included in the PRS may not be available in the validation data. In this case, it is important to report what proportion were available—and if a high proportion are missing it may be worth looking for proxies or considering a different PRS.

### 2.4 Aligning SNPs Between Base and Validation Data

We have previously mentioned that it is important to be aware of the genome build used in both the validation data and in the data within which the PRS was developed. There are a few other differences that are possible between genetic data-sets—they could have been typed using different genotyping platforms or arrays, with different strand orientations, or imputed using different software tools.

All of these things can result in slight differences in the way each SNP is labelled and presented, and it is important to ensure that the correct variants have been identified for inclusion in the PRS.

#### 2.4.1 Strand-Flipping

Since the betas of our PRS are an estimate of the effect of one allele (the “effect” or “risk” allele) of the SNP compared to the other (“non-effect” allele), it is important that the dosages we calculate are the number of copies of that effect allele. However, the alleles of any given SNP are not always given consistently between datasets. We illustrate five different situations in Table 4, and describe the methods needed to align or “harmonise” the data.

**TABLE 4:** Five examples of possible disagreements between PRS and validation data, when data harmonisation may be required. We illustrate five different situations in the table: Perfect agreement, labelling disagreement, strand flip, strand flip and labelling disagreement, palindromic (ambiguous) SNP.

		PRS summary data file		Validation data	
		Effect allele	Non-effect allele	Effect allele	Non-effect allele
1	Expected scenario - perfect agreement	A	C	A	C
2	PRS and validation data disagree on labelling of effect allele	A	C	C	A
3	“Strand flip”	A	C	T	G
4	Strand flip and labelling disagreement	A	C	G	T
5	Palindromic	A	T	T	A

One convention is for the less frequently occurring allele (minor allele) to be considered the effect allele, since it is a change from the population norm—under this labelling, an effect allele could be inversely associated with the condition of interest. An alternative approach is to label the alleles that increase risk of a condition as the effect alleles. Where two datasets have taken different approaches to this labelling, or when the less frequent allele changes between populations, the labels could be inverted between our data sets (see Row 2, Table 4).

When the effect and non-effect allele are inverted between datasets then this can be resolved automatically by some software (e.g., PLINK 2) or manually by relabelling the effect and non-effect allele in the PRS summary data, and inverting the effect size accordingly (since the effect size is the additive effect of each copy of the effect allele compared to the baseline of homozygous non-effect allele, we would multiply by -1 to obtain the inverse effect size).

A more complex situation arises when the datasets were genotyped using different DNA strand conventions. Although recent GWAS reports are almost always in reference to the forward strand as a consequence of imputation to a common reference panel, this is not always the case, and we may need to ensure that our datasets are harmonised prior to analyses (Hartwig et al., 2016).

If one dataset was genotyped in reference to the forward strand and the other in reference to the backward strand then the “backward” data would list the nucleotides that paired with the bases on the forward stand. Any instance of “A” on the forward strand would be “T” on the backward, “C” on forward would be “G” on backward and vice versa (see Rows 3 and 4, Table 4).

Some software (e.g., PRSice-2) can handle strand flips automatically, for others (eg PLINK 2) these will need to be identified and resolved manually.

#### 2.4.2 Ambiguous SNPs

Ambiguity arises when the SNP is palindromic (i.e., its alleles are nucleotides that pair with each other in a DNA molecule, such as A/T, see Row 5, Table 4). If the effect allele frequencies (EAFs) from the base data are available then we can compare them to the frequencies in our data and identify the alleles accordingly, but when the EAFs are close to 50% we cannot tell whether the effect and non-effect allele have been inverted, or whether the DNA strand is flipped. In these cases, or when allele frequencies in the base data are not available, then we cannot be certain about applying our weighting in the correct direction and should therefore exclude the SNP.

In PLINK 2, this can be achieved by first computing EAFs using the --freq command then filtering the output (.afreq) using awk to get a list of ambiguous SNPs [e.g., palindromic SNPs with EAF in the range 40 and 60% (Chen et al., 2018)]. Finally, the PLINK 2 command --exclude can be used to filter out the listed SNPs.

#### 2.4.3 Multi-Allelic SNPs

Multi-allelic SNPs have multiple possible alternate alleles for one reference allele, and these can be represented and identified in different ways in different data formats. In the UKB imputed data, these multi-allelic SNPs have been stored as a series of bi-allelic variants, sharing the same rsID and chromosome position and with the same listed reference allele but different alternate alleles.

The rsID and “chr:pos” identifiers are therefore not sufficient to uniquely identify one SNP, and allele information must be incorporated. This can be important during SNP extraction and PRS calculation, since we wish to ensure that we are including the correct alleles in our PRS calculation. In addition, many software tools require a unique identifier for each SNP. We discuss this further in the Online Materials.

#### 2.4.4 Compare Allele Frequencies

When the source data for the PRS makes effect allele frequencies available, then a good check is to compare the frequencies of these alleles in the validation data. This can be helpful not only for dealing with palindromic SNPs but also as a general sanity check.

While allele frequencies are unlikely to be identical between datasets, as the population will contain a different group of individuals and may be of different ancestries, it is reassuring if the frequencies are similar.

### 2.5 Quality Control

When using an existing PRS, it is important to first ensure that it is of good quality and is appropriate for the analysis data. Errors in genotype data can have many causes, including mix-ups or contamination of the samples, and malfunctions of the genotype probes. Without removing these errors, the resulting analyses may have reduced power and validity.

**FIGURE 2:**
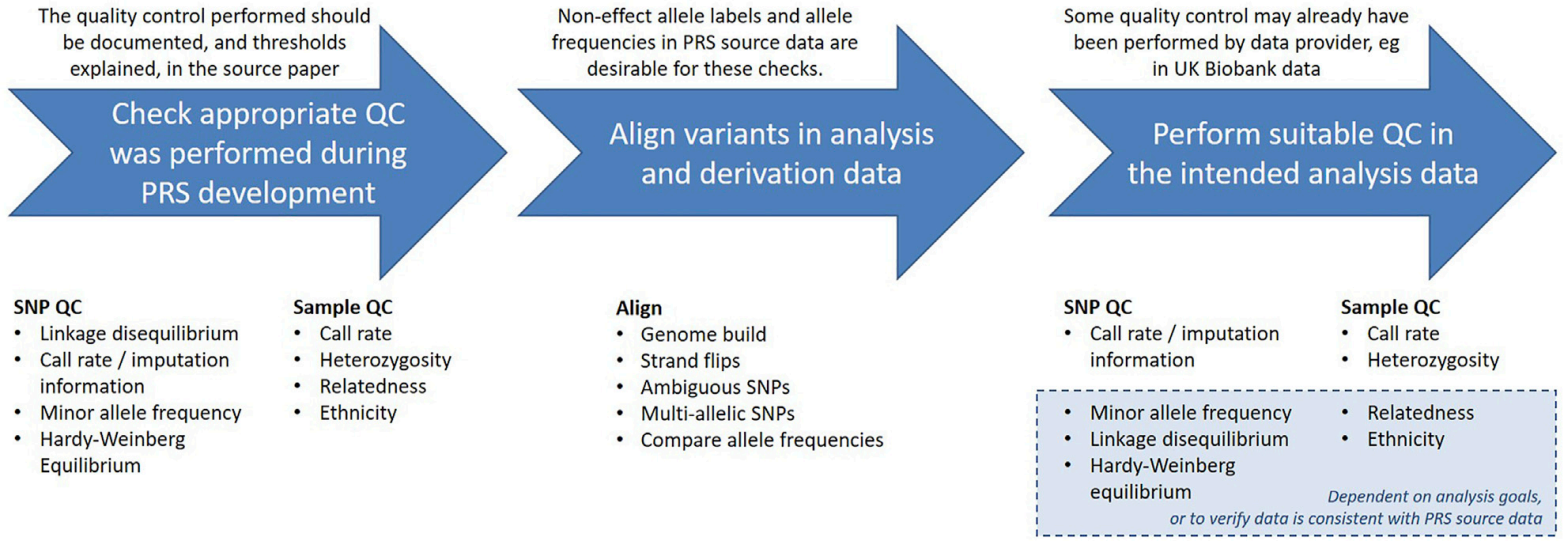
Summary of quality control and alignment steps.

There are a range of quality control considerations for genetic data that aim to identify and exclude potential data errors. In this section we will discuss these checks and indicate which may be relevant when calculating an existing PRS, outlined in Figure 2. The threshold values for many of these checks can be arbitrary and will vary depending on the purpose of the analysis, but we will give some examples from the literature.

The authors who developed the PRS should have provided documentation detailing the quality control (QC) performed on the base and target data, and being able to identify the steps taken is useful for determining if the PRS is suitable for the intended analyses. Since PRS are normally derived from GWAS summary statistics, the data will most likely have been subject to the typical GWAS QC checks, described in detail elsewhere (Reed et al., 2015; Marees et al., 2018; Choi et al., 2020).

Both the genetic variants included in the analysis (SNPs) and the individuals in the analysis population (samples) should have undergone these quality checks. A standard process could involve filtering at the SNP level first, followed by sample level filtering, and finally filtering SNPs based on Hardy-Weinberg equilibrium (HWE), as suggested by (Reed et al., 2015). The rationale for this is that HWE can be influenced by the population structure of the sample, and we will discuss this further in Section 2.5.3. Alternatively, sometimes SNP and sample filtering are iteratively applied with increasingly stringent thresholds (Marees et al., 2018).

In the case of imputed genotyping data these QC checks are typically performed on the directly called data prior to imputation, which means both that the imputation is conducted using high quality data, and that any lower quality data that was excluded may then be imputed. After imputation, the quality of each imputed variant is calculated, and those that were poorly imputed may then be excluded from further analyses. When using data that has already been imputed it may still be worth running further checks on the data, for example to use more stringent thresholds than were applied prior to imputation, depending on the intended analysis.

The focus of our discussion, the UK Biobank data, was genotyped by Affymetrix, who only provided genotype calls for SNPs and samples that satisfied their QC^8^. UK Biobank then applied a QC pipeline designed to accommodate both the large-scale, diverse population and the broad range of research questions the data would be used for, and made summary statistics available in the Data Showcase to facilitate further QC by researchers (Bycroft et al., 2018). These include variant-level statistics computed in QCTOOL for the imputed data (“Imputation MAF + info” files^9^) and downloadable variables (Category 100313, Genotyping process and sample QC^10^) which indicate lower quality samples.

#### 2.5.1 SNP QC

The SNP QC required during the development of a PRS is described in detail elsewhere (Reed et al., 2015; Marees et al., 2018; Choi et al., 2020), but we provide a brief overview to give a rough understanding of the rationale behind each check.

It is also important to ensure that the SNPs required for our chosen PRS are of sufficient quality in our intended analysis data. Any variants that were poorly genotyped in this data may warrant exclusion so they do not compromise the power of the score. We indicate which quality control metrics should be inspected when calculating an existing PRS, with examples for some that may be of situational interest.

##### 2.5.1.1 Linkage Disequilibrium

Linkage disequilibrium (LD) is a measure of the correlation between neighbouring genetic variants that are more likely to be inherited together because of their physical proximity, leading to association within a population. As in classic statistical modelling, multicollinearity can lead to problems with the model, and so any SNPs in high LD will typically have been identified and removed during the development of the PRS by methods such as “pruning” or “clumping” (Privé et al., 2019).

Since patterns of LD may vary among populations, particularly those of different ancestries, it may be of interest to verify that the SNPs in the PRS remain independent in the analysis data (Sawyer et al., 2005).

In addition, when calculating a PRS for a condition such as Alzheimer’s disease that has established high-risk variants (APOE e4), one may wish to exclude such variants from the polygenic score in order to consider them separately in the statistical modelling. In this case, we advise checking that no variants in the score are in LD with the high risk variant(s).

Details of how to investigate and filter on LD statistics using PLINK 2 can be found in the appendix of our online materials.^11^

##### 2.5.1.2 Imputation Information

Genotype imputation is the estimation of missing genotype calls by statistical inference. Increasingly, imputation is being used not only to fill in missing data caused by genotyping errors, but also to estimate the genotypes of variants that were not directly assayed, in order to increase the number of SNPs available in the data.

The “imputation information” statistic is a measure of imputation quality which typically takes values between 0 and 1, where 0 indicates complete uncertainty and 1 represents complete certainty about the imputed genotype. Depending on the software used, there are a different few information metrics that can be used to assess the quality of imputed data, but they are generally highly correlated (Marchini and Howie, 2010).

The UK Biobank carried out imputation on the genotype data using SHAPEIT3 and IMPUTE4 to statistically infer the genotypes of variants that had not been directly called in the genotyping array, and those which were missing or had been set to missing in central UKB quality control. They used QCTOOL (-snp-stats) to calculate the imputation information, and made it available to researchers in the “MAF + Info” files (UKB Resource 1967^12^). Bycroft et al. advise that “*An information score of α in a sample of M individuals indicates that the amount of data at the imputed marker is approximately equivalent to a set of perfectly observed genotype data in a sample size of αM*” and note that an information measure of 0.3 should yield good power to detect association given the large sample size of UKB (Bycroft et al., 2018).

If the PRS was developed on imputed data then the authors will normally have set a threshold imputation information score at which SNPs were eligible for inclusion, however it is possible that a variant that was well imputed in the base/target data was poorly imputed in the intended analysis data, so it is worth checking that all imputed SNPs in the score are good quality.

##### 2.5.1.3 Minor Allele Frequency

For a given SNP, the allele which is most common in the population is known as the “major” allele and the less common allele(s) are “minor.” The minor allele frequency (MAF) indicates how rare a variant is—typically a minor allele with frequency >5% is considered “common” while those between 1 and 5% are “low frequency” and MAF <1% is said to be “rare.”

If the frequency of the minor allele of a SNP is too low then we will not have adequate power to make meaningful statistical statements. Similarly when using imputed genotyping data, the imputation information of a SNP is likely to be correlated with its MAF, since there is less power available for imputing rare SNPs.

It is therefore common for SNPs with MAF below a certain threshold to have been excluded during GWAS and the development of PRS. The threshold for such exclusion varies depending on the aims of the original analysis and the size of the dataset - larger datasets give more power, and allow for the analysis of rarer variants.

Note however that the allele frequency is dependent on the population under study - for example some alleles are more common in individuals of particular ancestry. It is possible some SNPs will be rarer in the intended analysis data than in the data where the PRS was developed, in which case a decision must be made on whether to include them.

##### 2.5.1.4 SNP Call Rate

The call rate for a SNP is the proportion of individuals with non-missing data for that SNP. If a SNP has a low call rate then it may have been poorly assayed, and including it may result in spurious data (Turner et al., 2011). SNPs with a low call rate are therefore often excluded.

In the case of imputed genotype data, any assayed SNPs with call rate below a chosen threshold are generally considered poor quality and excluded prior to imputation. These excluded SNPs may then have their genotypes imputed, along with any missing calls in other SNPs, resulting in a complete data set.

#### 2.5.2 Sample QC

The word “sample” in this context refers to the individuals whose genetic data we are working with (like sample size in statistics). As with the genetic variants, the goal is to make sure that all individuals included in the study have high quality data, and the criteria considered during the calculation of the PRS are typically those used in GWAS.

When calculating an existing PRS, the QC again depends on the aims of the analysis. If it is an association analysis for example, evaluating the strength of association between the PRS and some trait or outcome of interest, then the focus is on the data at a population level, and exclusion of related individuals and restriction to a single ethnic group may be desirable, or included in sensitivity analyses. Alternatively, if the goal is to model how the PRS would perform if incorporated into clinical guidance, perhaps simulating a theoretical intervention to be offered at a given risk threshold, then one might wish to calculate the PRS for all individuals except those for whom there is reason to believe there were errors in genotyping.

Within the UK Biobank data, QC was performed to identify a subset of high quality, unrelated samples for use in the calculation of principal components. The details of the principal components analysis (PCA) are beyond the scope of this paper, and are described elsewhere (Bycroft et al., 2018). In short, UK Biobank used them to supplement the ethnic groups self-reported by participants and identify a group of individuals considered to be genetically of “White British ancestry” This White British ancestry subset is made available to researchers in UKB Data Field 22006.^13^

In addition, a directly downloadable variable (UKB Data Field 22020^14^) is provided which indicates whether a participant’s genetic data met the quality control checks required to be used in the calculation of these principal components (Bycroft et al., 2018). These checks comprised:

• Exclude individuals who were outliers for heterozygosity or missing rates.
• Exclude individuals with a missing rate >0.02 on autosomes.
• Exclude individuals with sex discordance (between the phenotypic and genetically inferred sex), or for whom genetic sex could not be determined.
• Exclude individuals who are not in a maximal set of unrelated individuals up to 3rd degree.

We will go through the rationale for each of these exclusions in the following sections.

##### 2.5.2.1 Heterozygosity

Heterozygosity is when an individual has two different alleles at a locus—an individual with the same allele on both chromosomes is homozygous at that locus. Heterozygosity is typically higher in individuals from mixed ethnic backgrounds, and lower in individuals whose parents are closely related. Extreme heterozygosity can indicate poor sample quality, and thus outliers are typically excluded.

The UK Biobank has done central checks and identified individuals which extreme heterozygosity that is not explained by ancestry. These outlying individuals, alongside those who were outliers for missing data (see “Sample call rate”) are listed in UKB Data Field 22027.^15^

##### 2.5.2.2 Sample Call Rate

The sample call rate is defined as the proportion of SNPs with non-missing data for this sample. This is analogous to the SNP call rate, but for individuals instead of SNPs. Individuals with a low call rate have a high proportion of missing genetic data, which could indicate poor quality.

In the UK Biobank central checks, individuals who were outliers for missingness prior to imputation were identified. These individuals, along with those who were outliers for heterozygosity are listed in UKB Data Field 22027.

##### 2.5.2.3 Sex Discordance

When the genotype inferred from the X and Y chromosomes doesn’t match that reported by the participant then this is known as sex discordance. Although it could be due to gender reassignment or sex-chromosome aneuploidy it could also indicate unreliable data and individuals with sex discordance are therefore generally excluded. The genetically determined sex of individuals in UK Biobank is made available in UKB Data Field 22001^16^ and can be compared to the gender reported at baseline, UKB Data Field 31^17^.

##### 2.5.2.4 Relatedness

If the data contains participants who are closely related then their genomes would be more similar than those of unrelated individuals, which can lead to biased estimations in population-level analyses. In the UK Biobank, kinship coefficients were estimated for all pair of individuals using KING software (Manichaikul et al., 2010), and a rough categorisation of relatedness is available in UKB Data Field 22021.

When excluding related individuals, note that only *n*-1 from every cluster of n related individuals needs to be removed in order for the remaining population to be unrelated. The UK Biobank Data Field 22020 restricts to a maximal subset of unrelated (to the 3rd degree) individuals who were not sex discordant or outliers for missingness or heterozygosity. This is the subset of participants that was used by UK Biobank to calculate the genetic principal components, and the algorithm by which they were selected is discussed in detail in (Bycroft et al., 2018).

Note that while for many analyses the subset identified by UK Biobank is adequate and convenient, it did not take disease status into account when removing related individuals. For rare outcomes it may be advisable to construct a new maximal unrelated subpopulation that preferentially retains individuals with the condition of interest.

#### 2.5.3 Hardy-Weinberg Equilibrium

The Hardy-Weinberg Equilibrium (HWE) is a principle that states that allele and genotype frequencies in a stable population without evolutionary influences will stay constant between generations. Deviation from HWE indicates that genotype frequencies differ significantly from their expected values which could indicate genotyping errors, such variants are therefore often excluded from analyses (Marees et al., 2018; Zhao et al., 2018). Note that HWE is sensitive to population structure if allele frequencies differ between subpopulations, so the population should be stratified by ethnicity prior to testing HWE.

In the UK Biobank genotyping data, variants were tested for HWE within each genotyping batch among individuals of homogeneous European ancestry (computed via PCA), and were set to missing at a threshold of *p* < 10^−12^ prior to imputation.

It is important to be aware that HWE is an assumption of many genotype imputation methods, including the IMPUTE2 program (Howie et al., 2009). If such methods have been used, it may then not be appropriate to test whether the resulting imputed variants conform to HWE.

The PLINK 2 command --hwe will filter out variants which deviate from HWE with a *p*-value beyond the given threshold (Wigginton et al., 2005; Graffelman and Moreno, 2013). Note that the HWE test used in PLINK 2 does not appropriately account for the uncertainty in imputed data (Shriner, 2011, 2013).

### 2.6 Calculating Dosages

Imputed genotypes are generally given probabilistically, rather than as discrete values. For example, for a particular SNP with alleles A and B, is represented in. bgen as the trio of genotype probabilities  ℙ(AA) , ℙ(AB) and ℙ(BB) for each individual.

A directly genotyped SNP will have probability 1 of one genotype and 0 for the others, but at an imputed SNP an individual might have, for example, a 90% probability of being homozygous for allele A (genotype AA) and a 10% probability of being heterozygous (genotype AB).

To calculate a PRS, we want to convert this information on genotype probabilities into a single number per SNP giving the “dosage” of the effect allele. We are assuming additive genetic effects, where the phenotypic expression increases for each copy of the effect allele.

There are two main ways of doing this - allelic or hard-call dosages. The method used should be reported to allow for replication of the PRS and any results.

#### 2.6.1 Allelic Dosages

The allelic dosages are real numbers, $dosage_{ij}\in \left[0,\;2\right]$ calculated as the expected number of copies of the effect allele

allelic dosage= 2ℙ(BB)+ ℙ(AB)

where A is the non-effect allele and B is the effect allele.

Although it is obviously not biologically plausible for an individual to actually have fractional copies of a variant, this provides a dosage value that incorporates some of the uncertainty of the imputed genotype calls.

See the PLINK 2 command --export A for exporting allelic dosage into a separate file, which can be read in R for easy inspection.

#### 2.6.2 Hard-Called Dosages

Hard-called, or thresholded, dosages are integer values, $dosage_{ij}\in \left\{0,\;1,\;2\right\}$ for SNP i in individual j , that are obtained by choosing a threshold value at which to round the expected (allelic) dosage to a whole number.

For example, if we set threshold as 0.1 in PLINK 2 using --hard-call-threshold 0.1, the hard-call dosage will be assigned as follows:

$$hardcall\;dosage=\;\{\begin{array}{c} 0\;if\;allelic\;dosage\;\in \left[0.0,\;0.1\right]\\ 1\;if\;allelic\;dosage\;\in \left[0.9,\;1.1\right]\\ 2\;if\;allelic\;dosage\;\in \left[1.9,\;2.0\right]\\ Missing\;otherwise \end{array}$$

While this provides us with data that looks the same as directly called genotypes, and can be stored in the same file formats, it is also losing information, and if we convert our entire dataset to hard-calls under a given threshold then we would not be able to recover our original information or change the hard-call threshold used.

Note also that once the genotype probabilities have been collapsed into a single expected dosage, we can get the same hard-call dosage value for two genotype probability trios that convey very different certainty about the underlying genotype (see Table 5).

**TABLE 5:** Hard-call vs. allelic dosages: genotype probability trios and allelic and hard-called dosages for 2 SNPs of a theoretical individual.

	P (AA)	P (AB)	P(BB)	Allelic Dosage (B)	Hard-call Dosage (B)
SNP1	0.22	0.50	0.28	1.06	1
SNP2	0.02	0.90	0.08	1.06	1

In this example, the individual has an allelic dosage of 1.06 copies of allele B for both SNPs, which would result in them being categorised as heterozygous when using hard-call dosages with a threshold of 0.1. However, their imputed probability of having the heterozygous genotype for SNP1 is much lower than it is for SNP2.

See the PLINK 2 command --import-dosage-certainty to use hard-called dosages and discard the values with low certainty.

### 2.7 Calculating the Polygenic Risk Score

While occasionally a risk score may be computed as the unweighted sum of effect allele dosages (“allele count model”), the most common approach is to weight each allele dosage by its effect size, as described in Eq. (1), and that is the method we will focus on here.

The actual calculation of a PRS is numerically straightforward and can be computed directly in any standard scripting language, such as R or SAS, as a matrix multiplication of SNP dosages per individual by betas per SNP. Recall that if the effect sizes in the PRS were given as odds ratios or hazard ratios, they will need to be log-transformed at this point.

However, for large scores it can be more convenient to use genetics tools such as PLINK 2, which uses the --score command to calculate linear risk scores for each individual and has some configuration options built in to handle missing data and standardisation of the score.

#### 2.7.1 Missing Genotype Data

Although this guide primarily deals with imputed genotype data and advocates the use of allelic dosages, we will briefly outline some of the techniques used to handle missing data in the calculation of a PRS.

Directly genotyped data, or imputed data that has been hard-called, may contain missing data and although individuals and SNPs with a high proportion of missingness are typically excluded as part of the quality control, there can still be some genotypes missing for some individuals.

One common approach to dealing with missing data for a SNP is to use the effect allele frequency in the population in place of the missing dosage for the individual (analogous to mean imputation in statistical analyses). This is the default approach in PLINK 2, but can be disabled by using the --no-mean-imputation modifier.

Alternatively missing genotypes can be ignored, and any SNPs for which an individual is missing a dosage value will not contribute to the score. In this case, it is advisable to find the average PRS per individual by dividing by the number of non-missing SNP dosages. This prevents scores of individuals with missing genetic data from being consistently lower than scores of individuals with complete data, which would result in bias towards lower risk.

Since each individual (i.e., sample) could be missing a different number of SNPs, each participant’s total PRS should be divided by their number of non-missing alleles; our averaged PRS is calculated as

$$PRS_{j}=\;\frac{\sum_{i}^{N}\beta_{i}*dosage_{ij}}{P*M_{j}}$$

where P is the ploidy of the individual (2 in this case since human autosomes are diploid), and $M_{j}$ is the number of non-missing variants observed for individual j .

This averaging approach is also the default in PLINK 2, and the resulting averaged PRS is output in the “<Score name>_AVG” column of a PLINK format sample score file (.sscore). If a non-averaged PRS is preferred, then the cols = scoresums modifier can be specified.

#### 2.7.2 Transforming the Polygenic Risk Score for Use in Analyses

Once the PRS has been computed, there are a variety of transformations that can be applied for either comparison to other scores or to produce easily interpretable results in analyses.

As the number of SNPs included in a PRS increases, so does the theoretical range of the score. For example, a hypothetical individual who was homozygous for all risk alleles (dosage = 2) could have a score of 20 for a 100 SNP PRS where all betas were 0.1, but a score of 200,000 for a 1,000,000 SNP PRS with betas of 0.1. This means we cannot directly compare the scores for PRS containing different numbers of SNPs.

In order to compare such scores we may therefore wish to average the total PRS by the number of SNPs which ensures a similar scale regardless of the number of SNPs used. Be aware, however, that by discarding the absolute value of the PRS, we compromise our ability to identify outliers, compare the PRS across samples, or detect the effect of natural selection (Choi et al., 2020).

For use in association studies, one common approach is to categorise PRS into percentiles for ease of interpretation. Often tertiles, quartiles, quintiles, or deciles are used, or the top 1% are compared to the middle quintile. This allows easy comparison of “high risk” individuals to “average” ones—especially given that there’s currently no well-established cut-off threshold to define a “high PRS” (Cupido et al., 2021).

In order to include a PRS as a continuous variable in regression models, it is often standardised to a normal distribution with mean = 0 and SD = 1, so that the effect in the model can be given in units of 1 SD of the PRS. This transformation also serves as a pre-processing step when combining multiple PRS into one. For example, we might wish to average PRS for similar traits (e.g., systolic blood pressure, diastolic blood pressure and pulse pressure) into one combined “blood pressure” risk score for analysis as demonstrated in (Pazoki et al., 2018), or construct a “meta” PRS combining multiple PRS for one trait across studies (Inouye et al., 2018).

The PRS is also generally kept as a continuous variable when it is incorporated in risk prediction models, as we see in (Elliott et al., 2020) (A. Lee et al., 2019). It is still necessary to assess the linearity assumption in the model building stage (i.e., linear association between PRS and outcome), as outlined in (Sun et al., 2021).

Each transformation has its own limitations, we advise readers to carefully choose one based on their analysis objective.

### 2.8 PRS in Statistical Models

One of the general statistical considerations when incorporating PRS in a model is to account for population genetic structures to avoid bias, which can be achieved by adjusting for genetic principal components (PC) in the model (Price et al., 2006) or by more advanced methods such as mixed models (Price et al., 2010). Typically, the first 10 genetic PCs are considered as possible confounders, this number is routine but arbitrary (Reed et al., 2015). Even when the analysis population is restricted to a single ethnic group, the genetic PCs can capture population structure that is not available in self-reported ethnicity. In UKB, the first 40 PCs are available for researchers to download under (Data Field 22009^18^) (Bycroft et al., 2018).

Similarly, bias can arise when the data was genotyped using different arrays or across multiple batches—which is increasingly common as the size of studies increases (Turner et al., 2011). It is therefore standard practice to adjust for genotyping array (Inouye et al., 2018). In UK Biobank the first ∼50,000 people were genotyped using the UK BiLEVE Axiom Array, while the rest of the cohort were genotyped using the UK BioBank Axiom Array. Genotyping was performed in 106 batches of about 4,700 individuals, using a custom genotype calling pipeline developed by Affymetrix. Information on both the array and batch number for each participant is made available for researchers (Data Field 22000^19^), and UK Biobank internal quality control of the data was performed within batches to account for any batch-level discrepancies.

## 3 Results

We have developed a pipeline that, when supplied with a list of SNPs and betas, can extract required SNPs, apply chosen QC and calculate a PRS using bgenix and PLINK 2. For the full code, and additional documentation of technical aspects, see Online Materials: PRS Pipeline on GitHub.^20^

### 3.1 Worked Example

We chose the PRS for low-density lipoprotein cholesterol developed by Klarin et al. (2018) in the Million Veteran Program data, because it is a relatively recent PRS that provides a comprehensive selection of SNPs in the context of the current literature. It consists of 223 lipid-associated SNPs with weights derived in the 2017 Global Lipids Genetics Consortium (GLGC) exome array analysis (Liu et al., 2017), in association analyses that were adjusted for sex, age, age squared and up to four principal components.

In addition, previous work has already been done applying this PRS within the UK Biobank (Trinder et al., 2020a; Trinder et al., 2020b) and these results have been returned to the UKB and made available, so we are able to validate our results against theirs.

The SNP list and betas for LDL-C were obtained from Supplementary Table 11 of Klarin et al., and were labelled under genome build GRCh37.75. The PRS is also available from the PGS Catalog with polygenic score ID PGS000115^21^ (Lambert et al., 2021).

#### 3.1.1 Validation Data

Our validation dataset is the UK Biobank (UKB), a prospective cohort study of ∼500,000 volunteers of middle and old age (40–69 years) in the UK. All UKB participants were genotyped, yielding directly called data for around 850,000 genetic variants. Variants that failed quality control were excluded, and data for a further ∼9 million genetic variants was then imputed. Variant IDs were assigned according to the Genome Reference Consortium Human Build 37 (GRCh37) reference genome (Bycroft et al., 2018), and the data was aligned such that the first allele given in the. bgen files is the reference allele on the forward strand (UK Biobank Resource 531^22^).

Note that individuals who have withdrawn from the UKB cohort have had their IDs replaced with negative numbers in the sample file. This maintains the order of the remaining IDs, so they still line up with the genetic data, but enforces exclusion of withdrawn participants, as they can no longer be joined to the phenotypic data.

In the GLGC exome array analysis where the weights for the LDL-C PRS were derived, LDL-cholesterol was measured in mg/dL, and therefore the weights $\beta_{i}$ represent the increase of LDL-C in mg/dL for each unit increase in dosage of $SNP_{i}$ . In the UK Biobank, LDL-cholesterol in mmol/L was measured in each participant at baseline, by blood samples taken for assays. We therefore convert the LDL-C measurements from mmol/L to mg/dL by multiplying by 38.67.

#### 3.1.2 SNP Extraction and Review of QC

We used bgenix (Band and Marchini, 2018) to extract SNPs for the PRS from UKB imputation data. All 223 variants were available in the UKB imputed genetic data, and there were no multi-allelic or ambiguous SNPs. We verified that the allele frequencies of the SNPs were similar (within 0.1 percentage point) in our data to those reported in the supplementary materials of (Klarin et al., 2018).

In the GLGC analysis where the weights were derived, the quality control conducted centrally across 73 contributing studies included removal of ambiguous variants, exclusion of variants with call rate <0.9 or HWE *p* value <1 × 10^−7^ (Liu et al., 2017). In the MVP data where the PRS was developed, the threshold values used for imputation information and minor allele frequency were 0.3 and 0.0003 respectively (Klarin et al., 2018).

**FIGURE 3:**
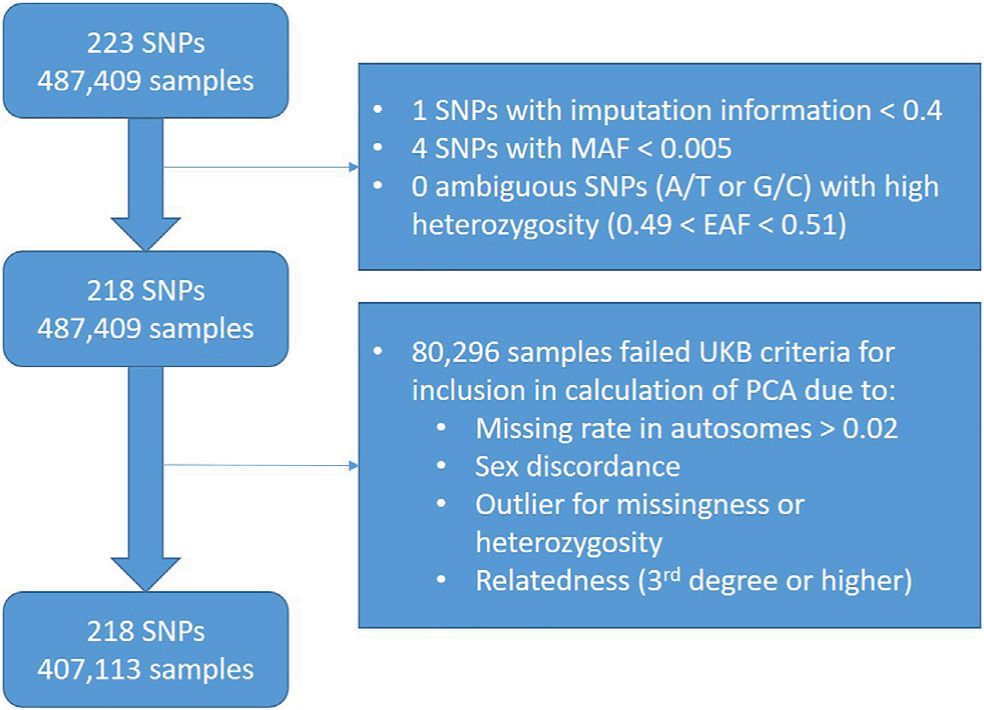
Flowchart showing quality control exclusions in worked example of LDL-C PRS in UK Biobank data.

We chose to exclude SNPs with an imputation information <0.4 within the UK Biobank data (*n* = 1), since this is a common threshold used in literature (Zheng et al., 2012). We also excluded rare SNPs with MAF <0.005 (*n* = 4). After these exclusions, we had 228 SNPs remaining (Figure 3).

**FIGURE 4:**
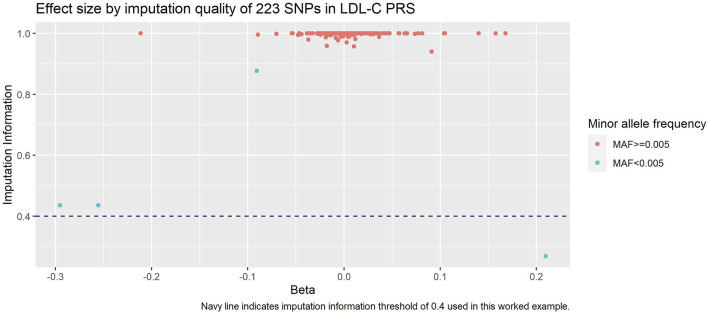
Imputation information against beta of each SNP in LDL-C PRS. Navy dashed line is our imputation information threshold of 0.4, and SNPs are coloured by our MAF threshold of 0.005.

When investigating the impact of these exclusions (Figure 4), we saw that the SNPs we excluded due to MAF included the SNPs with the lowest remaining imputation information - this is unsurprising since SNPs with lower MAF are generally less well imputed. In addition, we observed that these SNPs had some of the larger absolute effect sizes.

We excluded participants (*n* = 80,296) according to UK Biobank Data Field 22020, which indicates the subset of participants that met quality control for use in the calculation of principal components.

#### 3.1.3 Polygenic Risk Score Calculation and Validation

We calculated the PRS using allelic dosages in PLINK 2 with the cols = scoresums option to get the raw (non-averaged) values.

**FIGURE 5:**
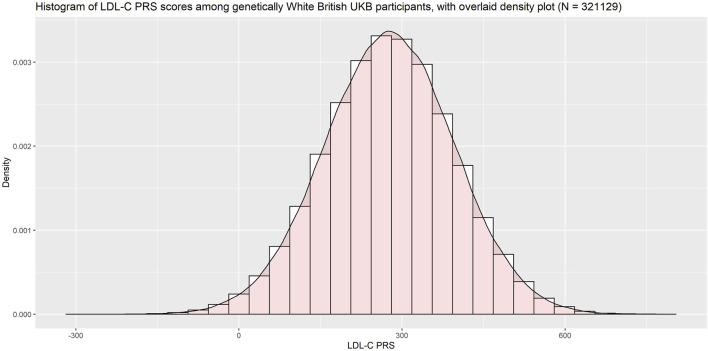
Histogram of LDL-C PRS with overlaid density plot.

Since the PRS was developed among primarily White individuals, we restricted our validation population to UK Biobank participants of genetically White British ancestry (using UKB Data Field 22006). Among this population the PRS was approximately normally distributed (Figure 5).

**FIGURE 6:**
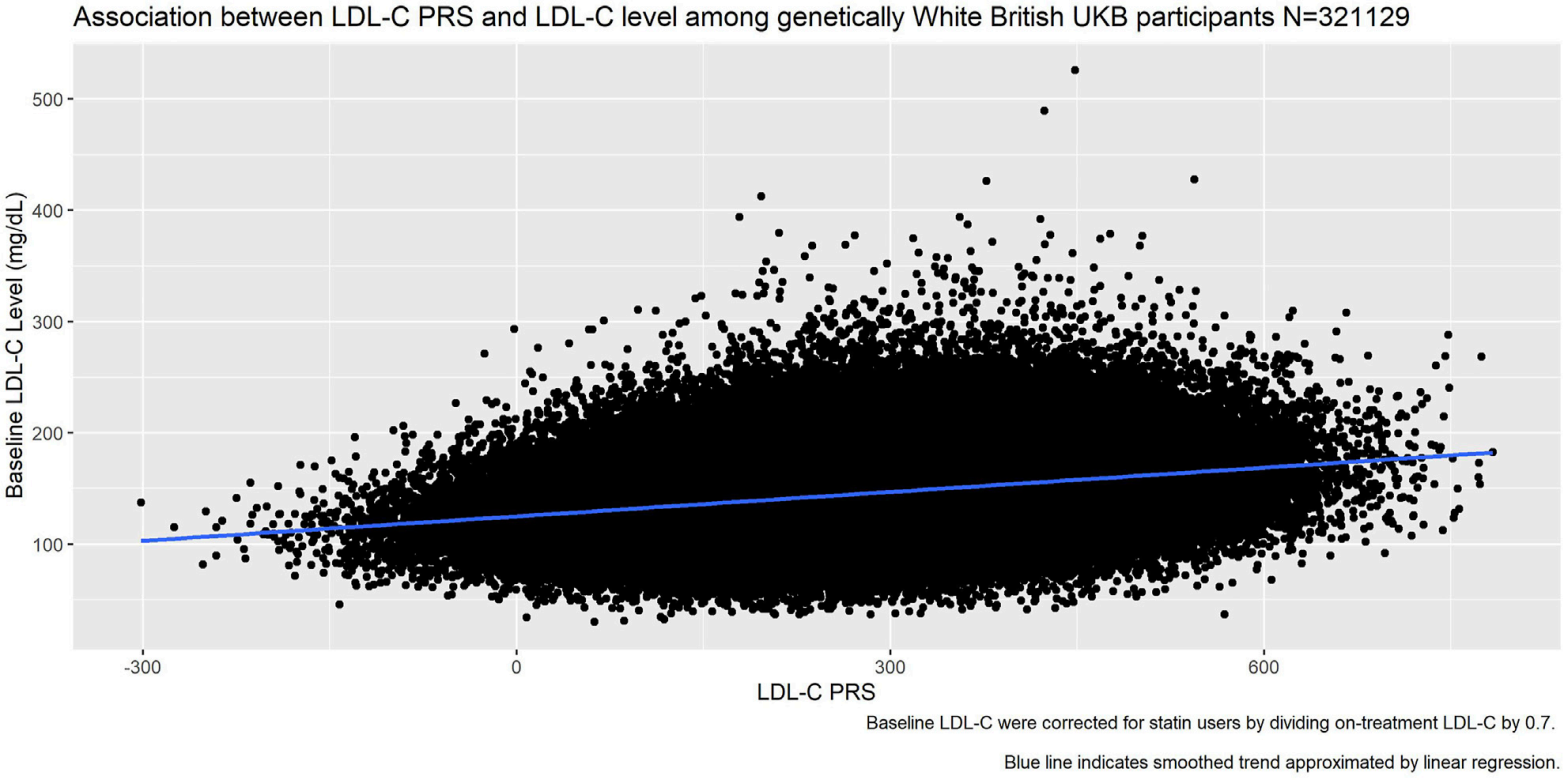
Association between LDL-C PRS and measured LDL-C at baseline among genetically White British UK Biobank participants.

Plotting the PRS against baseline LDL-C (Figure 6) we saw good association between the PRS and the measured LDL-C (*R* ^2^ = 0.27).

**FIGURE 7:**
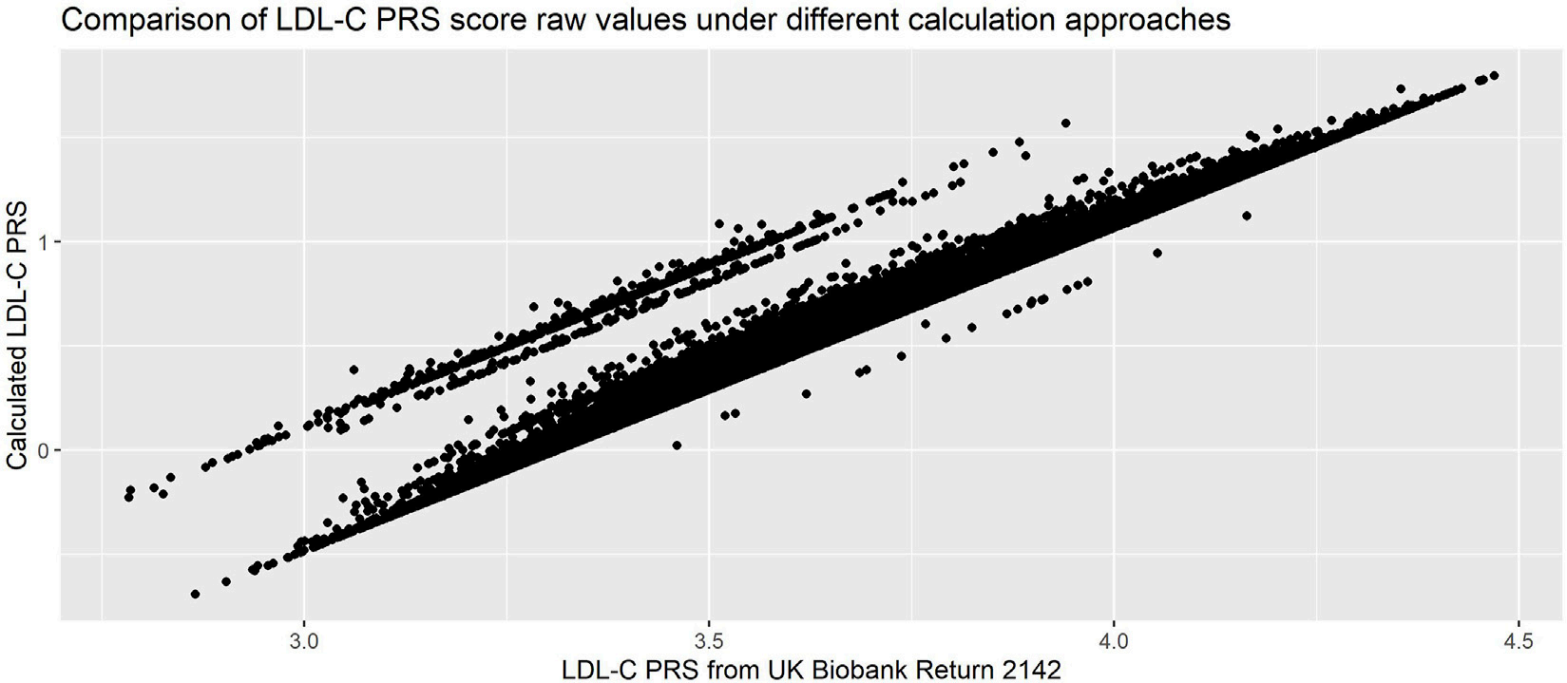
Comparison of PRS calculated using allelic and hard-call dosages (Pearson’s correlation coefficient = 0.99). PRS used was 223-SNP score from (Klarin et al., 2018), with hard-called dosage approach from (Trinder et al., 2020b).

We compared our calculated PRS with the one returned to the UK Biobank by Trinder et al. (UKB Return 2142^23^) and found almost perfect correlation (*R* ^2^ = 0.99). However, when inspecting a scatterplot of the scores (Figure 7) we observed differences in the raw values.

• We had allowed the betas to be either positive or negative, while in the calculation of the returned score all SNPs had been aligned such that the betas were positive. This resulted in our scores being consistently smaller.
• We had used allelic dosages, while the returned score had used hard-called dosages. This led to the parallel banding effect on the plot.
• Our quality control metrics differed slightly from those used in Trinder et al., leading to slightly different exclusions.

While both approaches are completely reasonable, the resulting scores are not directly comparable. This demonstrates the importance of carefully reading the methods used in the initial calculation of the PRS, in particular if the intent is to compare the performance or association in a new dataset with the initial publication.

### 3.2 Time and Computation Requirements

For this 223 SNP PRS, we ran each part with each of the three software tools discussed in this paper where possible, as a comparison we also ran a 118,388 SNP PRS for breast cancer (PGS000511^24^) (Fritsche et al., 2020). The computation times are presented in Table 6, and are not intended as an overall performance analysis of each tool, but rather as an indication of their relative speeds and scalability to larger datasets.

**TABLE 6:** Comparison of times taken. Please note absolute times may vary depending on the computation power of the system used, our interest is in the relative performance of the tools.

	bgenix	QCTOOL v2	PLINK 2
223 variants			
SNP extraction	53 s	2,696 s	18,403 s
QC	—	795 s	7 s
PRS calculation	—	—	1 s
100 k variants			
SNP extraction	2,681 s	>108 k s (exceeded 30 h limit)	20,821 s
QC	—	7,942 s	76 s
PRS calculation	—	—	256 s

Bgenix is the clear leader in terms of SNP extraction speed from BGEN files, as it was designed for this file format and takes advantage of the index file. While QCTOOL v2 offers a convenient wildcard feature to read from all the chromosome files in one command, it takes a long time to read the data and does not scale well to larger scores. PLINK 2 can rapidly extract data from its native. pgen file format, but in order to manipulate BGEN files it first auto-converts them to. pgen which takes approximately 25 min per chromosome on the full imputed data.

QCTOOL can calculate per-SNP or per-sample summary statistics quickly for small numbers of SNPs, but this scales poorly for large scores. In addition, some external tool (e.g., awk or R) is then needed to filter the resulting statistics by the desired exclusion thresholds, and then a separate extraction step must be used to apply these filters, which has not been included in our timings.

As previously discussed, PLINK 2 needs to convert the dataset to pgen format the first time it is read, but this only needs to be done once for a given score. Once the data has been converted, PLINK 2 can compute summary metrics and apply quality control thresholds in a single command, and does this rapidly even for large datasets.

Although the QCTOOL list of options includes the -risk-score command for PRS calculation, this is poorly documented and we have not explored it here. PLINK 2 can calculate even large PRS within a reasonable time.

## 4 Discussion

The continual hunt for “novel” variants associated with any given trait means new PRS are constantly being developed, using variants and effect sizes identified in GWAS conducted on ever-growing meta-analyses of multiple data-sets. This results in a wide array of scores for any given trait, with only minor improvements in predictive power beyond some threshold number of variants included.

However, the more data sets were used to contribute to the development of a PRS, the fewer datasets remain in which the score can be validated and used. We argue that there is value to be gained from using existing PRS in analyses, to validate and replicate the association and to investigate the potential for incorporating such scores in clinical practice. A PRS that has been incorporated in many analyses may become an “industry standard” score, and will result in more comparable research outputs than if many different scores were used.

Authors who develop PRS clearly hope that these scores will be used by others, and initiatives like the PGS Catalog and the Genetic Risk Prediction Studies (GRIPS) Statement have gone a long way towards making this possible by homogenising the reporting of the necessary information for replicating a PRS (Lambert et al., 2021; Wand et al., 2021).

Indeed, recent work (Becker et al., 2021) has made existing PRS even more accessible by arranging to make a selection of pre-calculated scores available for download within large datasets such as the UK Biobank. However, while this may offer a simple way for non-genetics focussed researchers to easily include PRS in their analyses, we should be wary that convenience does not overtake the need to critically evaluate the appropriateness of the score and the quality control applied.

In addition, even though the UK Biobank requests that all derived outputs are returned to them to be made available for other researchers to download, calculated PRS are not always returned and thus retrievable. Researchers who hope to use the same score are thus often obliged to reproduce the calculation, since direct sharing of UK Biobank data between studies is not permitted.

In this paper, we outlined the background concepts of PRS, compared genetic software tools for particular usage scenarios, and discussed the various QC metrics commonly used when working with genetic data, highlighting ways to best utilise resources provided by UKB. We provide our “PRS pipeline,”^25^ an easily modifiable and reusable script that takes an input file of betas and calculates the PRS.

In addition, we point out details which are often neglected in the reporting of existing literature but are crucial for reproducible work, such as different approaches to dosage computation. Finally, we discussed considerations of how PRS are computed and transformed to make sure they are appropriate for the research objective and statistical analyses.

### 4.1 Limitations

In this paper, we have focussed on the calculation of existing PRS for use in statistical analyses and modelling, and have not discussed techniques used to develop a new PRS or “real-world” applications of PRS in a clinical context. If PRS development is of interest, we recommend published guides for conducting GWAS and developing a PRS such as (Choi et al., 2020) and (Marees et al., 2018). Both provide online tutorials^26^,^27^ using either simulated or publicly available data (e.g., HapMap). Many applications have been proposed based on the analysis of PRS and these are discussed and showcased elsewhere, from exploring association of PRS with traits/outcomes, to assessing whether PRS improves existing risk prediction models (Elliott et al., 2020; Inouye et al., 2018; Lee et al., 2019; Sun et al., 2021), and investigating causal inference via Mendelian Randomisation (Klarin et al., 2018; Lewis & Vassos, 2020; Wray et al., 2021).

We also concentrated on the UK Biobank imputed data; while the methods we outlined are more generally applicable our assessment of the available software tools is specific to the BGEN v1.2 format. The UK Biobank is a large-scale, widely used cohort study, and is one of the most comprehensive genetic and health data resources currently available.

While the UK Biobank is launching a Research Analysis Platform (RAP) for online data access, the methods discussed in this paper will still be applicable for users who choose to download the data to work locally rather than incurring computation fees in the cloud. In addition, it is possible that the tools described in this guide may be made available on the platform.

## Data Availability

This research has been conducted using the UK Biobank Resource under Application Number 33952. Requests to access the data should be made via application to UK Biobank.

## Footnotes

1 https://www.pgscatalog.org/

2
https://prsweb.sph.umich.edu:8443/

3
https://2cjenn.github.io/PRS_Pipeline/

4
https://enkre.net/cgi-bin/code/bgen/doc/trunk/doc/wiki/bgenix.md

5
https://www.well.ox.ac.uk/∼gav/qctool_v2/

6 https://www.cog-genomics.org/plink/2.0/

7 https://www.pgscatalog.org/downloads/#scoring_columns

8 https://biobank.ndph.ox.ac.uk/showcase/ukb/docs/affy_data_generation2017.pdf

9
https://biobank.ctsu.ox.ac.uk/crystal/crystal/docs/ukb_genetic_data_description.txt

10
https://biobank.ndph.ox.ac.uk/showcase/label.cgi?id=100313

11
https://2cjenn.github.io/PRS_Pipeline/#Appendices

12
https://biobank.ndph.ox.ac.uk/ukb/refer.cgi?id=1967

13
https://biobank.ndph.ox.ac.uk/showcase/field.cgi?id=22006

14
https://biobank.ndph.ox.ac.uk/showcase/field.cgi?id=22020

15
https://biobank.ndph.ox.ac.uk/showcase/field.cgi?id=22027

16
https://biobank.ctsu.ox.ac.uk/crystal/field.cgi?id=22001

17
https://biobank.ctsu.ox.ac.uk/crystal/field.cgi?id=31

18
https://biobank.ndph.ox.ac.uk/showcase/field.cgi?id=22009

19
https://biobank.ndph.ox.ac.uk/showcase/field.cgi?id=22000

20
https://2cjenn.github.io/PRS_Pipeline/

21 https://www.pgscatalog.org/score/PGS000115/

22
https://biobank.ctsu.ox.ac.uk/crystal/ukb/docs/ukb_genetic_data_description.txt

23
https://biobank.ndph.ox.ac.uk/ukb/dset.cgi?id=2142

24 https://www.pgscatalog.org/score/PGS000511/

25
https://2cjenn.github.io/PRS_Pipeline/

26
https://choishingwan.github.io/PRS-Tutorial/

27
https://github.com/MareesAT/GWA_tutorial/
